# The effect of acupuncture on emotional disorders in patients with insomnia: A protocol for systemic review and meta-analysis

**DOI:** 10.1097/MD.0000000000023754

**Published:** 2020-12-18

**Authors:** Bi-Qing Huang, Gu-Xing Xu, Ling Luo

**Affiliations:** Acupuncture and Tuina School, Chengdu University of Traditional Chinese Medicine, Chengdu, China.

**Keywords:** insomnia, emotional disorders, acupuncture, anxiety, depression

## Abstract

**Background:**

Insomnia with high incidence is usually accompanied by many other diseases, especially mental disorders with the under-diagnosis and under-treatment. Some studies demonstrated that acupuncture may be effective for emotional disorders accompanied by insomnia. The systematic review protocol is designed to guiding analysis the effectiveness and safety of acupuncture for emotional disorders in patients with insomnia.

**Methods:**

Seven databases, Cochrane central register of controlled trials, Medline, Embase, China National Knowledge Infrastructure, Chinese Biomedical Literature database, VIP database and Wanfang database, will be searched from initial to December 2020. Randomized controlled trials (RCTs) of acupuncture for insomnia with emotional disorders (depression and anxiety) outcomes, which were reported in Chinese or English, will be included. The primary outcome is the change of degree of anxiety and depression. Study selection, data extraction and assessment of the risk of bias will be performed independently by 2 or more reviewers. Available data will be synthesized and statistically analyzed in RevMan V.5.3. The model of fixed effects will be used for the pooled data when the heterogeneity tests show little or no statistical heterogeneity (I2 < 50%). The random-effects model will be taken with heterogeneous data (50% ≤ I2 < 75%).

**Results:**

The effect of acupuncture on emotional disorders in patients with insomnia will be assessed on Hamilton anxiety Scale, Hamilton anxiety Scale, Pittsburgh Sleep Quality Index, Insomnia Severity Index, Self-rating Anxiety Scale, Self-rating Depressive Scale and the number of participants secede and the number of patients reported adverse events.

**Conclusion:**

the emotional disorders interaction with insomnia and the increase of risk on disease evolving and insomnia-related burden, it is so momentous to know that the role of insomnia treatment on comorbidities. We should concern about the management of emotional disorders when treat insomnia, and acupuncture treatment anxiety and depression caused by insomnia may be effective.

**Ethics and dissemination:**

Ethics approval is not be needed because the data will not contain individual patient data, and there are no concerns about privacy. The results of this meta-analysis will be disseminated through publication in a peer-reviewed academic journal or relevant conference.

**INPLASY registration number:**

INPLASY2020100115.

## 1 Introduction

Insomnia is characterized by difficultly falling asleep and staying asleep despite having enough opportunity to sleep.^[1]^ It occurs several times per week, result in significant distress and sleepiness, mood lability, etc.^[2]^. In the United States, the prevalence of insomnia disorder is 12% to 20%,^[3]^ and the insomnia symptoms in the general adult population ranges from 35% to 50%.^[4]^

Sleep is a major determinant of mental and physical health,^[5]^ however, more and more people are suffering insomnia. It is a risk factor for many disorders, especially emotional disorders.^[6–8]^ There are evidences indicate that chronic insomnia lead to anxiety and depression, and anxiety and depression will in turn worsen insomnia.^[9,10]^ Anxiety and depression are commonly comorbid with each other, with anxiety often temporally preceding the development of depression, anxiety may increase risk for the development of later depression through insomnia.^[11]^ Participants with insomnia, compared to those free of it, experienced more than double risk to develop depression.^[12]^ And even, anxiety and depression in turn will aggravate the symptoms of insomnia.^[13]^ A vicious circle formed between insomnia and emotional disorders. The emotional disorders are often ignored in treatment of insomnia. Moreover, owing to side effects and adverse event, the pharmacological therapies, such as selective serotonin reuptake inhibitors (SSRIs), are not the trusted approach to treat anxiety and depression.^[14]^

Acupuncture is among the most popular and safe procedures of the complementary and alternative medicine therapies,^[15]^ which is also a simple and useful treatment for improving insomnia.^[16–18]^ And some RCTs and systematic reviews have found that acupuncture are effectiveness and safety for depression and anxiety.^[19–26]^ The mechanism of acupuncture for anxiety may be the balance of prefrontal cortex (PFC) activity^[27]^ and endogenous melatonin secretion.^[28]^ The mechanism of acupuncture for depression is associated with the restoration of hippocampus Cornu Ammonis1(CA1) synaptic plasticity,^[29]^ increasing of adreno-cortico-tropic-hormone (ACTH) and neuropeptide Y,^[30–32]^ and reducing of the expression of 3 cytokines, interleukin-6, nerve growth factor, and tissue inhibitor metalloproteinase.^[33]^

## 2 Why it is significant to carry out this review

Given the emotional disorders interaction with insomnia and the increase of risk on disease evolving and insomnia-related burden, it is so momentous to know that the role of insomnia treatment on comorbidities. We should concern about the management of emotional disorders when treat insomnia, and acupuncture treatment anxiety and depression caused by insomnia may be effective. However, there is no systematic reviews to evaluate the efficacy of acupuncture in insomnia with emotional disorders. Therefore, a comprehensive review of acupuncture in insomnia patients with mood disorders will provide a useful analysis of overall effects of interrelated interventions, patients, practitioners and providers.

## 3 Objectives

The aims of this systematic review protocol are guide to obtaining a relatively persuasive conclusion of whether acupuncture is effective for treating emotional disorders in patients with insomnia.

## 4 Methods

The protocol has been registered in INPLASY **(**INPLASY2020100115), and completed according to the Preferred Reporting Items for Systematic Reviews and Meta-analysis protocol (PRISMA-P).^[34]^ Should any amendments to this protocol be necessary, they will be documented on the INPLASY platform.

### 4.1 Criteria for including studies in this review

#### 4.1.1 Types of studies

RCTs of acupuncture therapy for insomnia with anxiety or depression, which were reported in Chinese or English, will be included. The types of randomized including randomization numbers by random number table, envelope or other methods. Quasi-RCTs and uncontrolled clinical trials will be excluded.

#### 4.1.2 Types of participants

Patients were diagnosed primary insomnia, will be included regardless of the age, gender, source, and short-term or chronic insomnia in cases. The guideline of insomnia is unlimited, like International Classification of Sleep DisordersII/III (ICSD-2/ICSD-3)^[35,36]^ or Chinese guideline of insomnia disorder diagnosis and its treatment.^[37]^

#### 4.1.3 Types of intervention

The intervention of interest is needle stimulation of acupoints, including body acupuncture (manual/electro), auricular acupuncture and scalp acupuncture, and regardless of course and frequency of treatment.

#### 4.1.4 Comparator(s)/control

The comparators of the studies are unlimited but studies comparing different acupoints or different forms of acupuncture will be excluded.

### 4.2 Outcomes

#### 4.2.1 Primary outcomes

Emotional functioning outcomes scale: Hamilton Anxiety Scale (HAMA)^[38]^ to assess the severity of anxiety, Hamilton Depression Scale (HAMD)^[39]^ to evaluate the condition of depression.

#### 4.2.2 Secondary outcomes

Pittsburgh Sleep Quality Index (PSQI)^[40]^ and Insomnia Severity Index (ISI)^[8]^ are employed to assess the quality of sleep. Self-rating Anxiety Scale (SAS)^[20]^ and Self-rating Depressive Scale (SDS)^[20]^ are to evaluate the anxiety and depression separately. Adverse events, including the number of participants secede and the number of patients reported adverse events.

### 4.3 Search methods for identification of studies

#### 4.3.1 Searching from following databases

The databases will be searched from initiate to December 2020: Cochrane Central Register of Controlled Trials, Medline, EMBASE, China National Knowledge Infrastructure, Chinese Biomedical Literature Database, VIP Database and Wan fang Database. The following terms will be searched: insomnia, sleep, sleep disorder, sleep deprivation, sleep insufficient, acupuncture, manual acupuncture, electro-acupuncture, scalp acupuncture, and auricular acupuncture. The search strategy for MEDLINE is shown in Table 1. In the Chinese databases, the synonym items will be used.

**Table 1:** search strategy administrated in Medline database.

No.	Search items
#1	randomized controlled trial [pt]
#2	controlled clinical trial [pt]
#3	randomized [tiab]
#4	placebo [tiab]
#5	clinical trials [mesh]
#6	Trail[ti]
#7	#1 or #2 or #3 or #4 or #5 or #6
#8	Human [mesh]
#9	#7 and #8
#10	Insomnia [mesh]
#11	(insomnia, psychophysiological or psychophysiological insomnia or sleeplessness or sleep initiation dysfunctions or dysfunctions, sleep initiation or dysfunction, sleep initiation or sleep initiation dysfunction or insomnia, rebound or rebound insomnia or insomnia, transient or transient insomnia or insomnia, primary or primary insomnia or insomnia, nonorganic or nonorganic insomnia or awakening, early or early awakening or insomnia, chronic or chronic insomnia or insomnia disorder or disorders):ti, ab
#12	Sleep [mesh]
#13	(sleep or sleep disorder or sleep disturbance): ti, ab
#14	#10 or #11 or #12 or #13
#15	acupuncture therapy [mesh]
#16	(acupuncture or body acupuncture or manual acupuncture or electroacupuncture or electro-acupuncture or auricular acupuncture or laser acupuncture or warm needling or scalp acupuncture or acupoint): ti, ab
#17	#15 or #16
#18	#9 and #14 and #17

#### 4.3.2 Searching by other approaches

Clinical trial registries, like the WHO International Clinical Trial Registry Platform, Chinese clinical registry and Clinical Trials. Gov, will be searched for ongoing or have finished trials with unpublished data. The reference lists of all potential publications, including relevant systematic reviews, will be manually retrieved and reviewed to further locate additional trials. Incomplete data will be obtained by contacting the corresponding authors.

### 4.4 Collection and analysis of date

#### 4.4.1 Selecting studies

**Figure 1:**
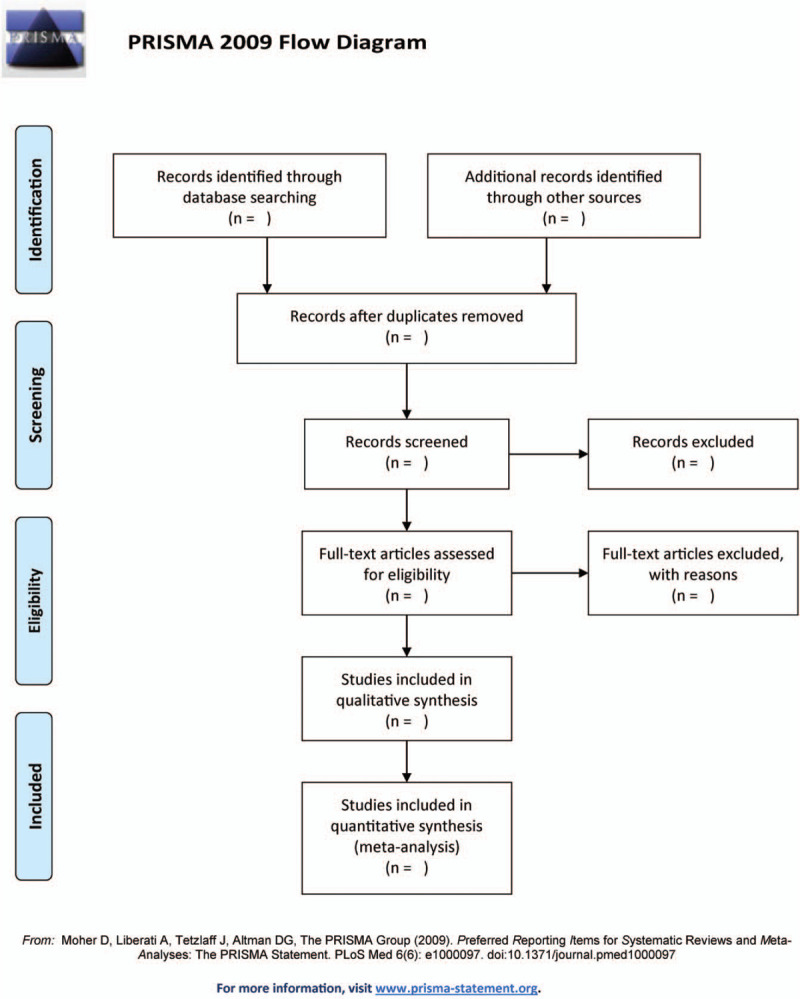
The Preferred Reporting Items for Systematic Reviews and Meta-Analyses diagram.

All of the titles and abstracts that we searched will be screened independently by 2 reviewers in terms of the inclusion criteria. The full text of eligible article will be reviewed if necessary. Any inconsistence will be arbitrated by another reviewer. Excluded studies will be listed in a table with reasons for their exclusion. The procedure of studies selection is shown in a Preferred Reporting Items for Systematic Reviews and Meta-Analyses diagram figure 1.

#### 4.4.2 Extraction and management of date

The following information will be extracted from all eligible studies by 2 independent reviewers (BQH and GXX) and entered a predefined data acquisition form: reference ID, author information, publication year, participant characteristics, intervention (acupuncture and controls), outcomes at all reported time points, and adverse effects, medication intake, duration of follow-up. Details about acupuncture techniques, like Number of needle insertions per subject per session, points, Needle types and retention time, will be extracted according to Standards for Reporting Interventions in Clinical Trials of Acupuncture (STRICA).^[41]^ Information not available from the studies will be obtained by contacting the corresponding authors. Any disagreements will be arbitrated by a third reviewer (LL). Cross-check of all data will be done by BQH and GXX, transferred into RevMan software (V.5.3).

#### 4.4.3 Evaluating risk of bias

The risk of bias in each study will be assessment by 2 or more independent reviewers using the Cochrane Collaborations tool for assessing risk of bias in randomized trials.^[42]^ The following 6 field will be assessed: selection, performance, detection, attrition, reporting bias and other sources of bias. Trials will be evaluated and divided into 3 levels: low, high risk, and unclear. Corresponding authors will be contacting for unclear items. Any disagreements will be arbitrated by a third reviewer LL.

#### 4.4.4 Dealing with missing data

The corresponding authors or relevant authors will be contacted by reviewers to obtain omissive data. An intention-to-treat analysis will be performed in the absence of lacking data. If possible, a sensitivity analysis will be administrated to address the potential impact of missing data, which will be addressed in the discussion if necessary.

#### 4.4.5 Assessment of heterogeneity

To investigating statistical heterogeneity in the forest plot, Q test will be applied with a significance level of *P* ≤ .1.^[43]^ Moreover, *I*^2^ test will be employed for quantifying inconsistency. The model of fixed effects will be used for the pooled data when the heterogeneity tests show little or no statistical heterogeneity in these trials (*I*^2^ < 50%). The random-effects model will be taken with heterogeneous data (50% ≤ *I*^2^ < 75%). Meta-analysis will not be conducted If there is considerable heterogeneity, and the qualitative analysis will be conducted.

#### 4.4.6 Data synthesis

Data synthesis will be conducted with clinical data by means of RevMan software (V.5.3). For dichotomous data, a risk ratio with 95% CIs will be applied for analysis. For continuous data, a mean difference or a standard mean difference (SMD) with 95% CIs will be employed for analysis. SMD will be applied if different assessment tools were used. We will apply the fixed-effects model to pooling data if the statistical heterogeneity is little or low, otherwise, the random-effects model will be performed.

#### 4.4.7 Assessment of publication biases

To observing the biases of potential reporting, funnel plots will be emerged when more than 10 studies are included.^[43]^

#### 4.4.8 Subgroup analysis and investigation of heterogeneity

If data are available, subgroup analysis will be performed. Variations in the characteristics, like the types of acupuncture and control intervention, short/long term effects, severity of anxiety and depression, will be considered.

### 4.5 Sensitivity analysis

A sensitivity analysis will be conducted to monitor the robustness and examine the potential influence through excluding studies from the analysis one by one. We will take care of some decision nodes, such as sample size, the weakness of methodological and missing data.

### 4.6 Summary of evidence

Two reviewers will assess independently the quality of evidence for each outcome by the Grading of Recommendations, Assessment, Development, and Evaluation system approach (GRADE),^[44]^ and present the result in “summary of findings” tables in the final report. “high”, “moderate”, “low” or “very low” grades will be rated in the assessments of data quality. The quality of evidence of a specific study will be assessed according to the risk of bias, imprecision, inconsistency, indirectness, publication bias, effect size or dose–response relation.

### 4.7 Ethics and dissemination

The approval of an ethical committee is not required to conduct this study. Patients and the public were not involved in the design and conception of this study. The results of this meta-analysis will be disseminated through publication in a peer-reviewed academic journal or relevant conference.

## 5 Discussion

This meta-analysis will evaluate whether acupuncture is effective for treating emotional disorders in patients with insomnia. This review will provide a useful analysis of overall effects of interrelated interventions, patients, practitioners and providers. If it is necessary to amend this protocol, we will provide the date of each amendment with statement of the changes and the corresponding reasons. This review will have potential limitations such as those related with the literature search strategy, since some potentially relevant documents (PhD dissertations, abstract of symposiums, etc.) might be missed and undermine the sensitivity of the search strategy; the language restriction to Japanese and Korean which might lead to language bias; and quantitative analysis may not be possible that different scales have been used in published trials.

## Acknowledgment

We gratitude to PhD Xiao-Chao Luo from the Chengdu University of Traditional Chinese Medicine for revising the draft of the manuscript.

## Author contributions

**Conceptualization:** Bi-Qing Huang, Gu-Xing Xu.

**Funding acquisition:** Ling Luo.

**Investigation:** Bi-Qing Huang.

**Supervision:** Ling Luo.

**Validation:** Bi-Qing Huang, Gu-Xing Xu, Ling Luo.

**Writing – original draft:** Bi-Qing Huang.

**Writing – review & editing:** Bi-Qing Huang, Gu-Xing Xu, Ling Luo.
